# Photobleaching alters the morphometric analysis of fluorescently labeled neurons and microglial cells

**DOI:** 10.3389/pore.2025.1612087

**Published:** 2025-04-04

**Authors:** Tamás F. Polgár, Krisztina Spisák, Zalán Kádár, Nora Alodah, Gabor J. Szebeni, Kata Klein, Roland Patai, László Siklós, Bernát Nógrádi

**Affiliations:** ^1^ Institute of Biophysics, HUN-REN Biological Research Centre, Szeged, Hungary; ^2^ Theoretical Medicine Doctoral School, University of Szeged, Szeged, Hungary; ^3^ College of Medicine, Alfaisal University, Riyadh, Saudi Arabia; ^4^ Laboratory of Functional Genomics, Core Facility, HUN-REN Biological Research Centre, Szeged, Hungary; ^5^ Department of Neurology, University of Szeged, Szeged, Hungary

**Keywords:** photobleaching, immunofluorescence, morphometry, morphology, fractal analysis

## Abstract

Photobleaching of immunofluorescence signal is a well-known phenomenon, however, its impact on derived parameters characterizing number and shape of different cell types in tissue sections is less understood. Our aim was to determine whether the duration of illumination and the type of fluorophore (Alexa Fluor 546 (A546), and Alexa Fluor 488 Plus (A488)) can influence the acquired morphometric parameters of cells in the nervous system. Immunofluorescent staining of microglia and neurons was performed on mouse spinal cord sections. Mean color intensity in a field of view, number of detectable neuronal cell profiles, partial coverage of microglial profiles, and fractal geometrical parameters were determined. All measurements were made using epifluorescence microscopy with identical acquisition parameters. Most of the measured parameters suffered significant alternation after 30–60 s of illumination. The data-altering effect of photobleaching was most prominent in the case of mean fluorescent intensity. Thus, while immunofluorescent staining is useful for co-localizing different groups of cells, cell-specific quantitative morphological measurements require photostable staining. Possibility of the combination of these methods on the same section in order to achieve multi-channel localization without photobleaching is exemplified.

## Introduction

Immunohistochemistry based on fluorescent immunostaining is now widely applied method to localize cellular components either in basic research or in clinical practice [1]. The method uses specific fluorescently labelled antibody, which binds to the antigen of interest, then its localization is determined by a fluorescent light microscope [2]. Compared to the alternative method, the chromogenic based immunohistochemistry, the immunofluorescence (IF) microscopy has an advantage of multiple staining, providing the simultaneous visualization of up to even 30 fluorophores [3]. Furthermore, recent development of the high-throughput multiplexed immunofluorescence method in combination with tissue clearing may provide the visualization and in depth localization of hundreds of proteins [4, 5].

Parallel to that the IF microscopy became widely used, the need to consider the quantitative aspects of the method emerged [6]. In such experiments, the amount and distribution of specific cellular components is aimed to be determined on the basis of the strength of the emitted fluorescent signal, for example, osterix and sclerostin during bone development, or chondroitin sulfate proteoglycans in perineuronal nets of motoneurons, or IgG and IgA in renal nephropathy [7–9]. However, such quantitative evaluation is rather challenging due to photobleaching, which is a phenomenon that occurs when fluorophores lose their ability to emit fluorescence upon prolonged exposure to excitation light, i.e., the fluorescent signal is fading over illumination time, and eventually disappear, thus may alter the quantitative data after image analysis [10]. Photobleaching is primarily attributed to the irreversible damage and chemical alterations incurred by fluorophores when subjected to high-energy excitation light [11]. This weakening in fluorescence may particularly affect the morphological characterization of objects with fine process arborization, for example, microglia and astrocytes. Microglia, the resident immune cells of the central nervous system (CNS), are the first line of defense against harmful events happening in the CNS, therefore the characterization of their size, shape and distribution is at the frontline of research attempting to understand the details of the degenerative processes affecting the brain tissue [12].

In research focusing on assessing immune/inflammatory events in diseases or in neuroprotection, measuring the morphological changes of these cells provides key information. For example, relative area coverage of microglia profile is routinely used to determine the level of microgliosis, indicating inflammation and possible neurodegenerative machinery in different anatomical areas as a populational answer for harmful events, while morphometric analysis on individual cell profiles offers a deeper insight into the quick changes in the central nervous system [13]. Such population-based area coverage measurements or individual microglia cell morphometry methods based on Scholl analysis or fractal geometry have a well-described literature, but as our repertoire of advanced methodology is growing, more detailed descriptions with new parameters to measure and new connections with function to map are being developed [14, 15]. As researchers extract more complex features from the cell profiles, the higher the chance of a measurement error by using suboptimal staining, biased measurement, or statistical methods that can easily lead to wrong conclusions. Regardless of the computational systems such as digital image processing methods, fractal geometry-based shape-descriptors, and artificial intelligence which are valuable tools to perform measurements, providing good quality and unmitigated image input remained the most basic and crucial factor. Our aim was to determine whether different illumination times (30–900 s) and fluorophores with different levels of photostability can alter the morphometric parameters measured on relatively simple shaped neurons and complex shaped microglia in the CNS. A combination of the fluorescent and the chromogenic labelling method, providing the advantages of the photostable marker for quantification and the fluorescent marker for identifying cell populations, is also presented.

## Materials and methods

### Animals and ethical considerations

Ethical approval for the animal experiments was given by (1) The Government Office in Csongrád-Csanád County, Hungary and (2) The Committee for Animal Experiments of the University of Szeged, Szeged, Hungary XVI./2733/2022. All experiments were carried out in accordance with the institutional guidelines for the use and care of experimental animals and the governmental law for animal protection (XXVIII. chapter IV. paragraph 31) which conforms to international laws and policies (EEC Council Directive 86/609, OJL 358 1 DEC. 12, 1987; NIH Guide for the Care and Use of Laboratory Animals, United States National Research Council, revised 1996). All efforts were made to minimize animal suffering.

### Sample preparation and immunolabelling

Under irreversible anesthesia with Avertin (Merck, Darmstadt, Germany), 10-week-old male Balb/c mice were transcardially perfused with 10 mM phosphate buffered saline (PBS; pH 7.4) followed by 4% paraformaldehyde (Merck) fixation in 10 mM PBS (pH 7.4). The lumbar spinal cord was removed and fixed overnight in the same fixative at 4°C. Then the samples were cryoprotected in 30% sucrose (Merck), dissolved in 10 mM PBS, for 1 day at 4°C and embedded in optimal cutting temperature (OCT) medium (Sakura Finetek, CA, United States). 30 μm thick consecutive sections were cut with a CM1860 cryostat (Leica, Wetzlar, Germany), and collected individually in 10 mM PBS in wells of tissue culture plates and stored at 4°C until processed for free-floating immunohistochemical staining.

For immunofluorescent labelling, sections were rinsed with 10 mM PBS (three changes, 5 min each), then blocked with 3% normal donkey serum (Sigma, St. Louis, MO, United States; Cat# D9663, RRID: AB_2810235) in 10 mM Tris-Phosphate Buffer Saline (TPBS) for 60 min was used to match the host species of the secondary labeling. All primary antibodies were marked either with donkey anti-rabbit Alexa Fluor 488 Plus with increased photostability (A488; Invitrogen, Waltham, MA, United States; Cat# A32790, RRID:AB_2762823) or Alexa Fluor 546 (A546; Invitrogen, Cat# A11056; RRID:AB_2534103) diluted to 1:400 in PBS for 60 min at room temperature. Afterward, sections were washed in 10 mM PBS (three changes, 5 min each), mounted on silane-coated glass slides (10-15 sections per slide), and covered with Fluoromount-G mounting media (Invitrogen, Cat# 00-4958-02) to match the refractive index and to minimize the effect of photobleaching. Lastly, samples were visualized under an Eclipse 80i microscope (Nikon) equipped with a Lumen 200 mercury lamp (Prior Scientific, Cambridge, United Kingdom).

For the combined diaminobenzidine (DAB) and fluorescent labelling, sections were sections were blocked and rinsed as routinely prepared in our laboratory. Then, a step was added to block the endogenous peroxidase activity with incubation in 0.3% hydrogen peroxide in 10 mM TPBS for 60 min. This was followed by overnight incubation at 4°C with a primary antibody cocktail containing a polyclonal rabbit-anti-rat antibody against Iba1 and a polyclonal sheep-anti-ChAT antibody (Merck, Cat# AB144P, RRID:AB_2079751) diluted to 1:250 and to 1:500, respectively, in 10 mM TPBS. After washing in 10 mM PBS (3 changes, 5 min each), sections were incubated at room temperature in a donkey-anti-sheep A488 fluorescent secondary antibodies for 1 h, dissolved in 1:100 dilution in 10 mM TPBS. Afterwards, non-specific stainings were blocked for 1 h with a mixture of 1%–1% normal goat (Vector Laboratories) and normal donkey serum (Sigma) in 10 mM TPBS, for 1 h, then sections were incubated in a biotinylated goat-anti-rabbit antibody. Next, all the sections were rinsed in 10 mM PBS (3 changes, 5 min each), incubated in the avidin-biotin complex diluted to 1:1600 in PBS for 1 h at room temperature. Next, after washing in 10 mM PBS, the reactions were visualized by incubation in 0.5% DAB as a non-bleaching staining (Merck, Cat#: 868272-85-9) with 1.5% NiCl_2_ in 10 mM PBS for 15 min. Finally, sections were washed in 10 mM PBS (3 changes, 5 min each), mounted on silane-coated glass slides, covered with Gel/Mount (Biomeda, Foster City, CA, United States).

### Image capturing and image analysis

To avoid photobleaching before capturing the first image, a dedicated section on the slide served as a control to set the imaging parameters of the microscope and the Micropublisher 5.0 RTV camera (Teledyne QImaging, Surrey, Canada), then uniformed image acquisition parameters (exposure time, acquisition gain, -gamma, -offset, -resolution) were used during capturing of whole time series. After setting the parameters, an area with uniformly distributed cells was selected and the focus was briefly adjusted before capturing the first image. Then a single image was taken after 30, 60, 120, 300, 600, and 900 s of illumination (without changing the parameters of the microscope or the imaging) to investigate the effect of fluorescent fading by measuring the following parameters: i) The mean color intensity of the whole microscopic images at different observation times and staining methods were measured with the Histogram module of the Photoshop program (Adobe, San Jose, CA, United States). ii) Changes in the numbers of detectable NeuN-positive cell profiles were determined: t cells which discernable nucleus were counted at each time point, and compared to the cell counts obtained at the initial image. Those cells with detectable nucleus at the initial time point, but no detectable nucleus at the observed illumination time point were considered “faded”. iii) The value of pixels forming microglia/macrophage cell profiles was determined using an automated macro module developed in our laboratory for the Image-Pro Plus (IPP) (Media Cybernetics, Rockville, MD, United States) image analysis software [13]. Relative differences in the values obtained at different observation times and staining methods were measured to express the effect of fluorescent fading using the background parameters obtained in the initial image. iv) Fractal geometrical parameters were measured according to the work of Fernández-Arjona and her colleagues [15]. Sections were screened under the microscope at ×20 magnification with a Plan Fluor 20x/0.50 DIC N2 objective (Nikon). Microglial cells were identified and captured at different observation times and staining. The segmentation requires a certain amount of detail visibility. For this an endpoint of detection was determined based on mean color intensity; cell profiles from A488 staining were segmented after 0 and 900 s of illumination, while cell profiles from A546 staining were segmented after 120 s of illumination. Individual cell profiles completely in frame (one image contained 2-5 microglia), with visible soma and branch structure, clearly distinguishable from the surrounding cells were selected and converted to a binary image, then continuity errors were corrected manually using Photoshop (Adobe) and ImageJ (National Institute of Health, Bethesda, MA, United States). From the segmented microglia profiles, cell area, cell perimeter, fractal dimension, lacunarity, span ratio, density, and circularity were extracted by the Box Counting Fractal Analysis method using the FracLac plugin of ImageJ [16].

In general, higher fractal dimension means higher complexity; in the case of microglia morphometry, fractal dimension helps to identify intermediate microglia phenotypes. Lacunarity describes the objects rotational and translational invariance; changes in the soma can alter lacunarity. Cell area and perimeter are pixel-based parameters used to map cell size changes, while span ratio, density, and circularity are size-invariant descriptors of the phenotype, and tools to measure morphological changes.

All measured parameters serve a standalone way of comparison between photostable standards and also between antibodies with “regular” and “enhanced” photostability.

### Statistical analysis

To determine the average intensity loss, the number of NeuN-positive neurons and the relative changes in the microglial area 15-15 sections were imaged from 0 s to 900 s of illumination. Altogether 90 individual profiles, two from each image, were selected and segmented from these sections (n = 15/group) and were pooled according to the fluorophore and illumination time. To measure the effect of fading during the illumination linear regression was also conducted using the stats statistical package in Python (version 3.10.9, python.org) on signal intensity loss measurements. Before each comparison, Shapiro-Wilk normality test was used, followed by the appropriate parametric/non-parametric statistical test to assess the differences among multiple means of intensity loss, the number of neurons, relative changes of microglial coverage and changes in the fractal parameters. In each case, the statistical test is specified in the figure legends. All data are presented as mean values ± the standard error of the means (s.e.m.). All statistical analyses were performed in Prism (version 8.0.1.244, GraphPad Software, San Diego, CA, United States).

## Results

### Changes in mean signal intensity

**FIGURE 1:**
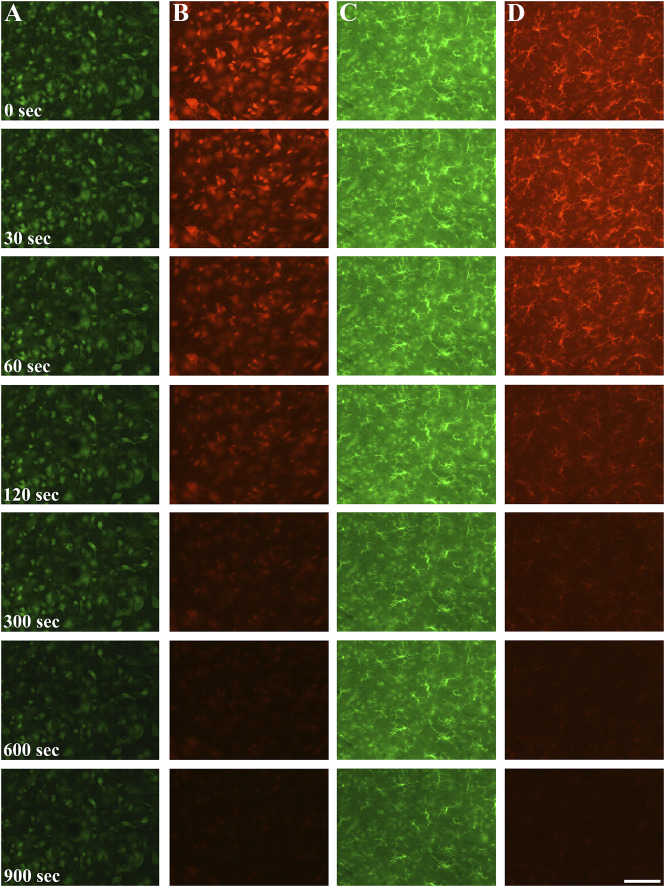
Neuronal (column **(A, B)**) and microglial (column **(C, D)**) staining using Alexa Fluor 488 Plus secondary antibody (column **(A, C)**) and Alexa Fluor 546 secondary antibody (column **(B, D)**) at 0 s exposition, after 30, 60, 120, 300, 600 s and 900 s excitation times. Qualitatively, both of the fluorophores fading commenced as early as after 60 s illumination time, and by 900 s only faint staining (A488) if any visible signal (A546) is noticed. Scalebar: 100 μm.

**FIGURE 2:**
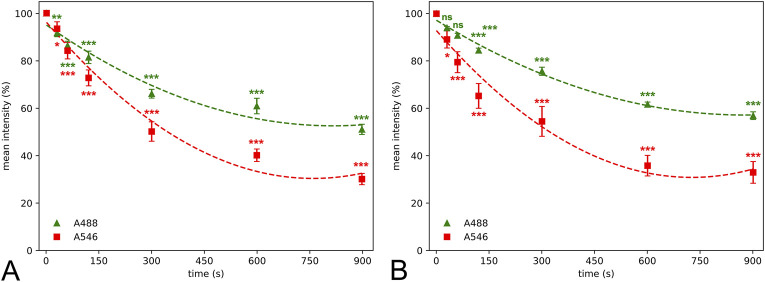
Quantitative determination of mean color intensity revealed that photobleaching lead to significant decrease of the fluorescent light as early as after 30–60 s compared to values at 0 s illumination time of microglial **(A)** and neuronal staining **(B)**. Alexa Fluor 546 (A546) shows a higher mean intensity loss dynamic than Alexa Fluor 488 Plus (A488) resulting in a significant difference at 90 s illumination time and 60 s time points in case of microglial and neuronal staining, respectively. Statistical evaluation was carried out using parametric one-way analysis of the variance (A488 neuronal staining, A546 microglial staining) and Kruskal-Wallis test (A546 neuronal staining, A488 microglial staining) with the Dunn’s multiple comparison test (*: p < 0.05, **: p < 0.01, ***: p < 0.001, ns: not significant). Second-degree polynomial curve was fitted to estimate the temporality of photobleaching.

Fluorescent fading was first assessed qualitatively in large field of view images after neuronal and microglial staining using A488 and A546 fluorophores (Figure 1). Although the bleaching dynamics of the fluorophores were somewhat different, we found significant data loss as early as 30 s of illumination time in the case of the neuronal staining (Figure 2). The intensity of both fluorophores was reduced by more than 10% after 60 s, and the intensity loss was doubled after 120 s of continuous illumination, regardless of the primary antibodies. The two secondary antibodies showed a different photobleaching dynamic. By the 900 s illumination time, since mean signal intensity represented the whole image, some background intensity remained, but almost all microglia and neuron profiles disappeared. At the end of the illumination series, A488 showed a 45% decrease compared to the initial intensity; furthermore, in the case of the A546 fluorophore, this intensity loss reached 65%.

### Changes in the number of detectable neurons

**FIGURE 3:**
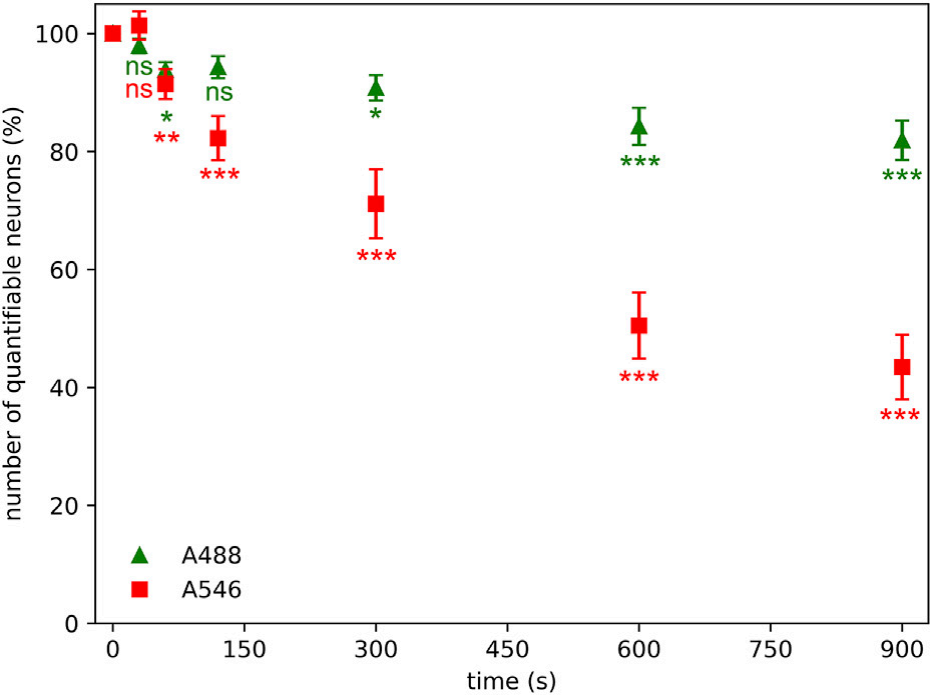
Photobleaching has a significant effect on quantifiable neuronal number (stained neurons with discernible nucleus) even after 60 s of illumination time using Alexa Fluor 546 (A546) and Alexa Fluor 488 Plus (A488) compared to the values measured at zero sec illumination time. Statistical evaluation was determined using Kruskal-Wallis test with Dunn’s multiple comparison test (*: p < 0.05, **: p < 0.01, ***: p < 0.001, ns: not significant).

Neurons in the sections were counted in the same area with different illumination times if their nuclei could be identified. The number of detectable neuronal profiles was reduced by approximately 10% after 60 s of illumination of samples, regardless of the fluorophore (Figure 3). The number of neuronal profiles faded relatively slowly if samples were stained with A488 fluorophore; however, the photobleaching was more prominent in the case of A546, where the decrease in the number of NeuN-positive profiles after 120 s of illumination reaches 20%. At the end of the experiment, A488 showed relatively good preservation, with a 22% decrease in the number of detectable neurons, but more than half of the initial number of neuronal profiles disappeared if samples were stained with A546.

### Changes in relative area coverage of microglial cells

**FIGURE 4:**
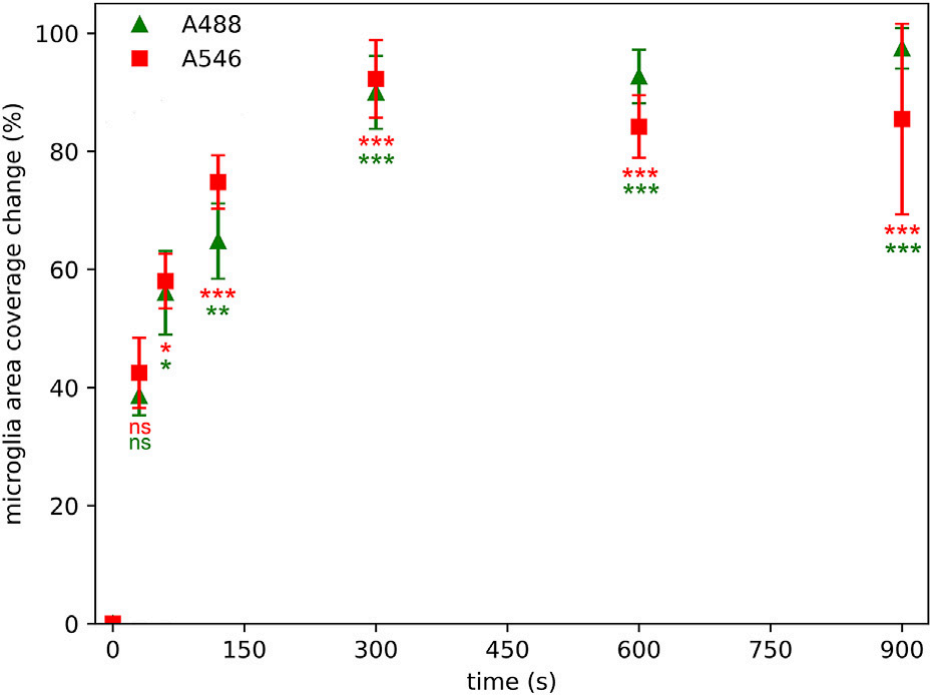
Photobleaching has a significant effect on microglial area coverage measurements even after 60 s of illumination time using Alexa Fluor 546 (A546) and Alexa Fluor 488 Plus (A488) compared to area coverage measured at zero time point. Statistical evaluation was determined using Kruskal-Wallis test with Dunn’s multiple comparison test (*: p < 0.05, **: p < 0.01, ***: p < 0.001, ns: not significant).

The relative microglial cell profile area coverage measurement maps the background density and standard deviation of the background to provide a cut-off value of the image of interest for segmentation. The same area at different time points was segmented and the relative differences between the actual image and the initial image was calculated. Notable changes occurred already after 60 s of illumination (Figure 4). Even after such a brief illumination, approximately 55% changes in the relative area occupied by microglia were observed regardless of the fluorophores. This change scaled up to a 90% loss after 300 s of illumination regardless of the fluorophore.

### Changes in fractal parameters

**FIGURE 5:**
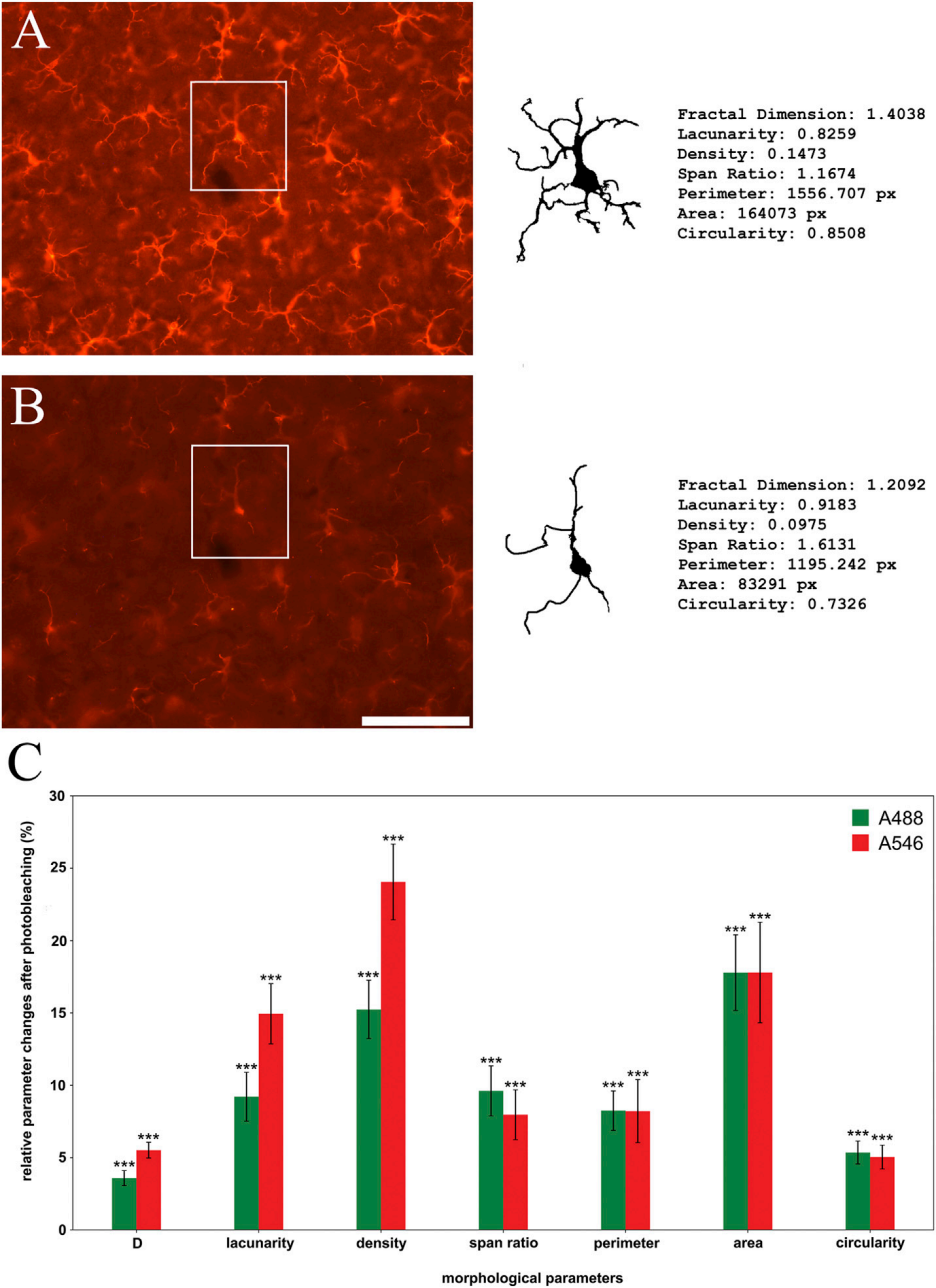
Photobleaching has a significant effect on morphological parameters of individual microglial cell profiles (**(A)**: initial image, **(B)**: faded image) using Alexa Fluor 546 (A546; red bars on panel **(C)**) and Alexa Fluor 488 Plus (A488; green bars on panel **(C)**). Qualitative analysis reveals significant changes of all observed parameters **(C)**. Statistical evaluation was determined using the one sample t-test (A546 density, A546 fractal dimension) or Wilcoxon Signed Rank Test (all other parameters) on data retrieved at the point of detection limit (900 s of illumination in the case of A488 and 120 s in the case of A546). *p* < 0.001. D: Fractal dimension. Scalebar: 100 μm.

Size-, outline-, and shape-based morphometric parameters were compared between individual cell profiles segmented from microscopic images taken at different time points (Figure 5). Based on the mean intensity changes and considering the time-consuming process of semi-manual segmentation cell profiles were segmented at the 0-time point and at a determined endpoint of illumination time where the binarization process still could be done. This time was 120 s for A546 and 900 s for A488. The most prominent changes were observable in two of the cell-sized-based parameters (area (18% loss in the case of both conjugates) and density (15% loss in the case of A488% and 24% in the case of A546)), but the shape-based parameters also suffered significant information losses showing 4% (A488) and 6% (A546) change in fractal dimension, 9% (A488) and 15% (A546) in lacunarity, 10% (A488) and 8% (A546) in span ration, 8% (both conjugates) in perimeter and 8% (both conjugates) in circularity. In conclusion, pooled fractal parameters showed 10% (A488) and 12% (A546) information change at the endpoint of detection.

### Combination of the DAB-Based and the fluorescent based immunohistochemistry

**FIGURE 6:**
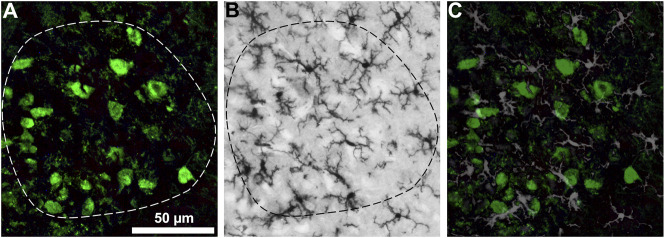
Hypoglossal nucleus double stained for motor neurons **(A)** and microglial cells **(B)**. The fluorescently stained motor neurons are used to identify the border of the hypoglossal nucleus **(A)**, which borderline is copied to the DAB-stained microglial stained image **(B)**, and the microglial area coverage could be determined within this border. For illustration of the co-distribution the two images are overlaid **(C)**. Scale bar: 50 μm.

For a reproducible morphological quantification within identified anatomical structure the DAB-based and the fluorescent based immunohistochemistry in a single section was combined. This method combines the advantages of the photostable DAB-staining for quantification and the cell-type specificity of the fluorescent staining. For illustration purposes, sections from the hypoglossal nucleus were used, since this motor nucleus displays easily identifiable borders compared to that of the ventrolateral motor neurons. In Figure 6, the fluorescently stained motor neurons are used to identify the border of the hypoglossal nucleus, which borderline is then copied to the DAB-stained microglial stained image, and the microglial area coverage could be determined within this border. For illustration of the co-distribution, the two images are overlaid.

## Discussion

The physical-chemical process of photobleaching is a well-described phenomenon, however, its effect on the trustworthiness of measured morphological data of biological samples is less investigated [17, 18]. In this study, the data-altering effect of photobleaching was examined on various quantification approaches to microglial and neuronal parameters in the CNS. Photobleaching has not only severely affected the fine morphological details of microglial cells, but also impacted the more rudimentary intensity- and area-based measurements and even neuronal cell counting. The various parameters were affected to an increasing extent in a time-dependent manner, with mean signal intensity and area coverage already showing altered parameters after 30 and 60 s of illumination, respectively.

Mean signal intensity measurement of immunofluorescent stainings is a widely used method for the quantification of protein expression [19]. Even with highly standardized settings (arc lamp intensity, detector settings, imaging features), due to the required pre-imaging adjustments (e.g., focus/z-range settings, region of interest selection), there is an unavoidable variability in illumination time, even with the exclusion of user-to-user and staining-to-staining variability. In our experiments, mean intensity was one of the most sensitive parameters affected by photobleaching, already showing significant data loss at the earliest measurement point (30 s of illumination). In practical settings, this short timeframe is comparable to the delays occurring due to the above-mentioned pre-imaging settings, suggesting that this widely used approach is inherently biased.

Another widely used quantification method is neuronal cell count, which is often applied as a primary readout parameter in characterizing CNS pathologies or evaluating the efficacy of therapeutic interventions [20]. While this method is generally viewed as a basic, yet robust technique, we also observed significant data loss regarding the number of identifiable neuronal cells. On the other hand, with the A488 secondary antibody, roughly 80% of neuronal cells remained identifiable even at the latest time point, suggesting that with sufficient optimization, this approach can be less affected by photobleaching, especially in comparison to intensity- and area-based methods.

**FIGURE 7:**
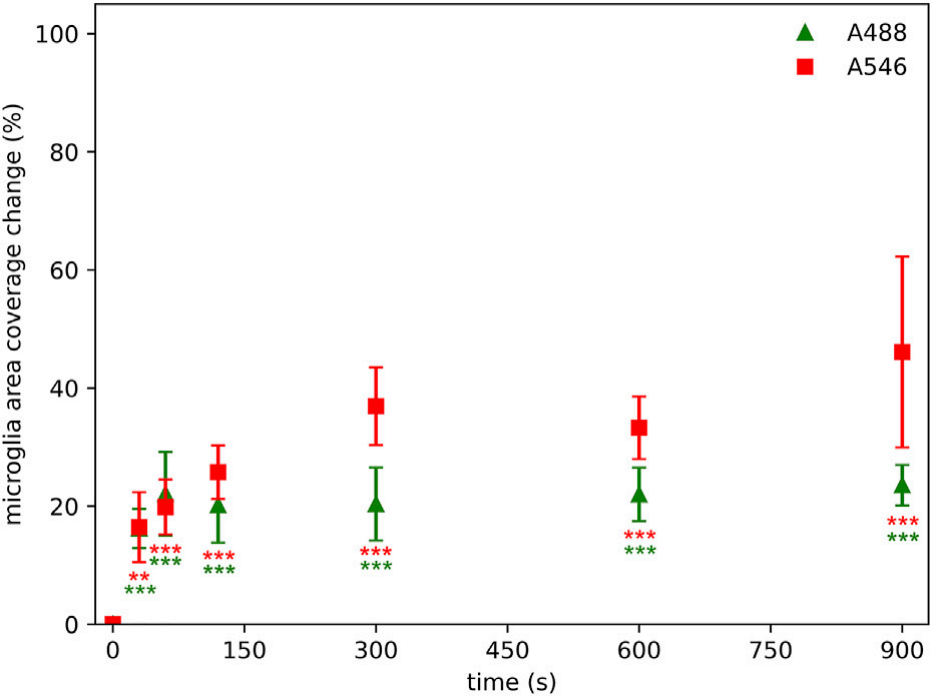
Photobleaching has a significant effect on microglial area coverage even after 30 s of illumination using Alexa Fluor 546 (A546) and Alexa Fluor 488 Plus (A488) compared to area coverage measured at zero time point, however, the information losses are not strictly monotonically increasing by illumination time. Lower time point acquisitions are representing an “expert” in the technical settings of the microscope or the anatomy of the particular sample, while higher illumination times representing an unexperienced user. Statistical evaluation was determined using Kruskal-Wallis test with the Dunn’s multiple comparison test. **: p < 0.01, ***: p < 0.001.

While intensity- and cell counting-based approaches are also used to characterize microglial activity in the CNS, these do not encapsulate the change in microglial morphology–a key feature of microglial activation [21]. Thus, as a rudimentary approach to quantify microglial morphology, area-based analysis is often utilized. Our results show that the area coverage of microglial cells was already reduced by 55% at the second measurement point (60 s), indicating drastic data loss. Consequently, fine microglial morphological parameters (that are derived from the segmented cell profiles–which are closely linked to the area coverage of the staining) were also affected by photobleaching. The primary aim of quantifying these fine morphometric parameters is to provide a more detailed, in-depth characterization of microglial cells, as they are able to detect subtle alterations of microglial structure and network, which can be further utilized for microglial clustering and phenotype analysis [22]. However, due to the unique and largely irregular shape of microglial cells and branches, these images are often captured and processed as z-stack projections. This means an even longer illumination period, which will affect the retrievable data to an even greater extent. Furthermore, the illumination time during examination can vary based on the expertise of the person performing the microscope parameter setting and the image acquisition. In case of an unexperienced user, or unfamiliarity with the particular sample and anatomic structure, the potential examination time cannot be predicted–along with the degree of information loss. This is represented by the dynamic area coverage change, where lower illumination time reflects an expert, while higher illumination times represent a more unexperienced user (Figure 7).

In neuroscientific research, in order to increase the resolution and image quality, and to better characterize the microglial network, laser-based imaging systems are often utilized. As the illumination intensity is often higher in scanning confocal and especially in super-resolution techniques, the effect of photobleaching on the derived parameters characterizing the number and shape of different cell types can be even more prominent than what we report here, with a traditional arc lamp epifluorescent solution [23]; however, the comparison between different imaging platforms and mounting media is warranted in the future. Nevertheless, our results regarding rapid loss of mean fluorescent intensity even after 30 s of illumination strongly suggest a similar quantification bias on other imaging systems as well.

In our experimental setup, we detected significant information loss relatively early in all attributes and conjugates, however, the dynamics of the information loss were different between the two commonly used fluorescent conjugates, A488 and A546. This difference might stem from the different features of the secondary antibodies, as in the case of the A488 antibody, the Alexa Fluor Plus reagent is expected to yield superior signal-to-noise ratio, in comparison to the conventional Alexa Fluor reagent (A546). Even though in this study we did not include additional secondary antibodies, literature suggests that photobleaching in far-red, carboxyfluorescein diacetate succinimidyl ester, CellTrace Violet, and CellVue Claret markers is a general phenomenon and affects wide variety of methods [24, 25]. Nevertheless, selection and optimization of the secondary antibody can not only improve the quality of the staining but might impact or mitigate the effect of photobleaching as well.

Reducing photobleaching in general has as wide of a literature as the tips and recommendations found on the information websites of the producers of the markers, but the issue is far from being completely solved [26, 27]. In our study, we gave an example of a combination of fluorescent and photostable information visualization that can be used with multiple fluorophore labeling for localization and a DAB-based solution for morphological evaluation. Moreover, we focused only on the impact of photobleaching on two major cell types of the CNS, as changes in population and individual microglial cell morphology and the number of neuronal cells are important and widely investigated attributes in CNS pathologies [22, 28]. The results based on the “bulky” appearance of the neurons and the “bushy”, fine structure of microglia indicate that our conclusions might also be applied to other cell types. In order to mimic routine experiments using IHC and epifluorescent imaging, we only used one illumination power–the one that was most optimal for the first section on the slide; however, investigating the effect of illumination power on the degree of photobleaching could provide further insight into the dynamics of photobleaching. Nevertheless, the suggestions based on our results can be extrapolated to any experiment where intensity, cell number, cell area, or fine morphological measurements are being done, as photobleaching universally affects various tissues and stainings.

Photobleaching significantly altered the retrievable data used to characterize microglial and neuronal morphology in the central nervous system. While some of the quantification methods were less affected by photobleaching (e.g., neuronal cell count and certain fractal parameters), more rudimentary techniques (e.g., area coverage and intensity measurement) were drastically altered by fluorophore degradation. Some of these changes were already evident after 30 and 60 s of illumination time, suggesting that this bias effect the majority of imaging techniques and users. “Mimicking” unexperienced and professional users showed the instability caused by photobleaching, while the fitted curves on the intensity loss plots show the temporality of photobleaching. The combination of photostable and fluorescent labelling is a potential alternative to classical multiplex fluorescent labelling, which can overcome this data-altering issue.

## Data Availability

The raw data supporting the conclusions of this article will be made available by the authors, without undue reservation.

## Abbreviations

Iba1, Ionized calcium-binding adaptor molecule 1; NeuN, Neuronal Nuclei; A488, Alexa Fluor Plus 488; A546, Alexa Fluor 546; DAB, diaminobenzidine tetrahydrochloride; PBS, Phosphate Buffered Saline; TPBS, Phosphate Buffered Saline with 0.2% Triton X-100; CNS, Central Nervous System; IPP, Image-Pro Plus; OCT, optimal cutting temperature.

## References

[1] Joshi S Yu D. Immunofluorescence. In: Basic science methods for clinical research. Houston, TX: Academic Press (2017). p. 135–50. Chapter 8. 10.1016/B978-0-12-803077-6.00008-4

[2] Galati DF Asai DJ. Immunofluorescence microscopy. Curr Protoc (2023) 3(8):e842. 10.1002/cpz1.842 37540554

[3] Bolognesi MM Manzoni M Scalia CR Zannella S Bosisio FM Faretta M Multiplex staining by sequential immunostaining and antibody removal on routine tissue sections. The J Histochem Cytochem : official J Histochem Soc (2017) 65(8):431–44. 10.1369/0022155417719419 PMC5533273 28692376

[4] Cho W Kim S Park YG. Towards multiplexed immunofluorescence of 3D tissues. Mol Brain (2023) 16(1):37. 10.1186/s13041-023-01027-9 37131224 PMC10155431

[5] Tan WCC Nerurkar SN Cai HY Ng HHM Wu D Wee YTF Overview of multiplex immunohistochemistry/immunofluorescence techniques in the era of cancer immunotherapy. Cancer Commun (London, England) (2020) 40(4):135–53. 10.1002/cac2.12023 PMC7170662 32301585

[6] Haaijman JJ. Immunofluorescence: quantitative considerations. Supplementband (1988) 35:77–83. 3138723

[7] Watanabe T Tamamura Y Hoshino A Makino Y Kamioka H Amagasa T Increasing participation of sclerostin in postnatal bone development, revealed by three-dimensional immunofluorescence morphometry. Bone (2012) 51(3):447–58. 10.1016/j.bone.2012.06.019 22766096

[8] Ritok A Kiss P Zaher A Wolf E Ducza L Bacskai T Distribution and postnatal development of chondroitin sulfate proteoglycans in the perineuronal nets of cholinergic motoneurons innervating extraocular muscles. Scientific Rep (2022) 12(1):21606. 10.1038/s41598-022-25692-3 PMC9751140 36517521

[9] Moldoveanu Z Suzuki H Reily C Satake K Novak L Xu N Experimental evidence of pathogenic role of IgG autoantibodies in IgA nephropathy. J Autoimmun (2021) 118:102593. 10.1016/j.jaut.2021.102593 33508637 PMC7997636

[10] Bernas T Robinson JP Asem EK Rajwa B. Loss of image quality in photobleaching during microscopic imaging of fluorescent probes bound to chromatin. J Biomed Opt (2005) 10(6):064015. 10.1117/1.2136313 16409080

[11] Rost FWD. Quantitative fluorescence microscopy. Cambridge: Cambridge University Press (1991). p. 115–28.

[12] Ransohoff RM Perry VH. Microglial physiology: unique stimuli, specialized responses. Annu Rev Immunol (2009) 27:119–45. 10.1146/annurev.immunol.021908.132528 19302036

[13] Paizs M Engelhardt JI Siklós L. Quantitative assessment of relative changes of immunohistochemical staining by light microscopy in specified anatomical regions. J Microsc (2009) 234(1):103–12. 10.1111/j.1365-2818.2009.03146.x 19335461

[14] Chiu KB Lee KM Robillard KN MacLean AG. A method to investigate astrocyte and microglial morphological changes in the aging brain of the rhesus macaque. Methods Mol Biol (Clifton, N.J.) (2019) 1938:265–76. 10.1007/978-1-4939-9068-9_19 PMC6428051 30617987

[15] Fernández-Arjona MDM Grondona JM Granados-Durán P Fernández-Llebrez P López-Ávalos MD. Microglia morphological categorization in a rat model of neuroinflammation by hierarchical cluster and principal components analysis. Front Cell Neurosci (2017) 11:235. 10.3389/fncel.2017.00235 28848398 PMC5550745

[16] Karperien A Ahammer H Jelinek HF. Quantitating the subtleties of microglial morphology with fractal analysis. Front Cell Neurosci (2013) 7:3. 10.3389/fncel.2013.00003 23386810 PMC3558688

[17] Hirschfeld T. Quantum efficiency independence of the time integrated emission from a fluorescent molecule. Appl Opt (1976) 15(12):3135–9. 10.1364/AO.15.003135 20168404

[18] Wüstner D Christensen T Solanko LM Sage D. Photobleaching kinetics and time-integrated emission of fluorescent probes in cellular membranes. Molecules (Basel, Switzerland) (2014) 19(8):11096–130. 10.3390/molecules190811096 25076144 PMC6271172

[19] Shihan MH Novo SG Le Marchand SJ Wang Y Duncan MK. A simple method for quantitating confocal fluorescent images. Biochem Biophys Rep (2021) 25:100916. 10.1016/j.bbrep.2021.100916 33553685 PMC7856428

[20] Coggeshall RE Lekan HA. Methods for determining numbers of cells and synapses: a case for more uniform standards of review. The J Comp Neurol (1996) 364(1):6–15. 10.1002/(SICI)1096-9861(19960101)364:1<6::AID-CNE2>3.0.CO;2-9 8789272

[21] Reddaway J Richardson PE Bevan RJ Stoneman J Palombo M. Microglial morphometric analysis: so many options, so little consistency. Front neuroinformatics (2023) 17:1211188. 10.3389/fninf.2023.1211188 PMC10448193 37637472

[22] Nógradi B Meszlényi V Patai R Polgár TF Spisák K Kristóf R Diazoxide blocks or reduces microgliosis when applied prior or subsequent to motor neuron injury in mice. Brain Res (2020) 1741:146875. 10.1016/j.brainres.2020.146875 32389588

[23] Vangindertael J Camacho R Sempels W Mizuno H Dedecker P Janssen KPF. An introduction to optical super-resolution microscopy for the adventurous biologist. Methods Appl fluorescence (2018) 6(2):022003. 10.1088/2050-6120/aaae0c 29422456

[24] Lacour W Adjili S Blaising J Favier A Monier K Mezhoud S Far-red fluorescent lipid-polymer probes for an efficient labeling of enveloped viruses. Adv Healthc Mater (2016) 5(16):2032–44. 10.1002/adhm.201600091 27113918 PMC7159338

[25] Zolnierowicz J Ambrozek-Latecka M Kawiak J Wasilewska D Hoser G. Monitoring cell proliferation *in vitro* with different cellular fluorescent dyes. Folia Histochem cytobiologica (2013) 51(3):193–200. 10.5603/FHC.2013.0027 24203624

[26] Bernas T Zarebski M Dobrucki JW Cook PR. Minimizing photobleaching during confocal microscopy of fluorescent probes bound to chromatin: role of anoxia and photon flux. J Microsc (2004) 215(Pt 3):281–96. 10.1111/j.0022-2720.2004.01377.x 15312193

[27] Ghithan JH Noel JM Roussel TJ McCall MA Alphenaar BW Mendes SB. Photobleaching reduction in modulated super-resolution microscopy. Microscopy (Oxford, England) (2021) 70(3):278–88. 10.1093/jmicro/dfaa062 33064828 PMC13396269

[28] Polgár TF Meszlényi V Nógrádi B Körmöczy L Spisák K Tripolszki K Passive transfer of blood sera from ALS patients with identified mutations results in elevated motoneuronal calcium level and loss of motor neurons in the spinal cord of mice. Int J Mol Sci (2021) 22(18):9994. 10.3390/ijms22189994 34576165 PMC8470779

